# Australians with metabolic dysfunction‐associated steatotic liver disease have a twofold increase in the incidence of cancer

**DOI:** 10.1002/jgh3.70000

**Published:** 2024-07-22

**Authors:** Elizabeth E Powell, Shruti Roche, Babak Sarraf, Gunter Hartel, Richard Skoien, Barbara Leggett, James O'Beirne, Patricia C Valery

**Affiliations:** ^1^ Department of Gastroenterology and Hepatology Princess Alexandra Hospital Woolloongabba Queensland Australia; ^2^ Centre for Liver Disease Research, Faculty of Medicine the University of Queensland Brisbane Queensland Australia; ^3^ QIMR Berghofer Medical Research Institute Herston Queensland Australia; ^4^ Royal Brisbane and Women's Hospital Herston Queensland Australia; ^5^ Department of Gastroenterology and Hepatology Royal Brisbane and Women's Hospital Herston Queensland Australia; ^6^ Faculty of Medicine, The University of Queensland Woolloongabba Queensland Australia; ^7^ Department of Gastroenterology and Hepatology Sunshine Coast University Hospital Sunshine Coast Queensland Australia

**Keywords:** cirrhosis, diabetes, extrahepatic cancer, liver disease

## Abstract

**Background and Aim:**

Metabolic dysfunction‐associated steatotic liver disease (MASLD) is associated with an increased risk of extrahepatic morbidity. We compared the incidence of cancers in adults admitted to Queensland hospitals with MASLD with that for the Queensland population and examined the association between cirrhosis and type 2 diabetes and the development of extrahepatic cancers.

**Methods:**

In this retrospective study, we identified all cancers (Queensland Cancer Registry) after the first hospitalization with MASLD during Jul‐2007 to Dec‐2019, estimated age‐standardized incidence (ASI) of cancers, and compared that with the ASI in the Queensland population (incidence rate ratios [IRR]). Among the MASLD cohort, we examined the association between diabetes and cancer risk (Cox regression). Median follow‐up was 3.8 years (54 204 person‐years).

**Results:**

Totally 1104 new cancers were diagnosed in 1018 patients (8.9% of 9771 non‐cirrhotic and 1712 adults with cirrhosis). The ASI (all cancers) of 1668.2 per 100 000 person‐years in men (95% CI 1523.7–1827.4) and 1284.0 per 100 000 person‐years in women (95% CI 1169.6–1408.2) was 2‐fold higher than that of the Queensland population (IRR = 1.94, 95% CI 1.75–2.16 and IRR = 1.99, 95% CI 1.78–2.22, respectively). Incidence of stomach cancer, unknown primary, and pancreas was 3‐ to 5‐fold higher compared to the general population (all *P* < 0.001). In multivariable analysis of the MASLD cohort, older age (e.g. ≥70 years adjusted hazard ratio [adj‐HR] = 4.59, 95% CI 3.61–5.83), male gender (adj‐HR = 1.20, 95% CI 1.05–1.37), and cirrhosis (adj‐HR = 1.37, 95% CI 1.11–1.70) were independently associated with extrahepatic cancer risk, while diabetes was not.

**Conclusions:**

Our findings will help to raise awareness among clinicians about the importance of cancer vigilance in this patient group.

## Graphical abstract

Incidence of cancers in adults admitted to Queensland hospitals with metabolic dysfunction‐associated steatotic liver disease (MASLD) during 2007‐2019 was 2‐fold higher than that of the Queensland population. Incidence of stomach cancer, unknown primary, and cancer of the pancreas was 3‐ to 5‐fold higher compared to the general population. Our findings will help to raise awareness among clinicians about the importance of cancer vigilance in this patient group.

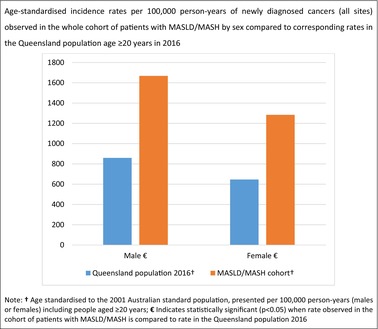

## Introduction

Although the association of metabolic dysfunction‐associated steatotic liver disease (MASLD) with primary liver cancer is well established, *extrahepatic* cancer (EHC) is far more common. It is the second leading cause of death in people with MASLD (after cardiovascular disease),^1^ and some,^2^, ^3^ but not all studies,^4^ report a higher risk (or incidence) of EHC in this patient population. In a meta‐analysis including over 180 000 individuals (one‐quarter with MASLD, mostly from Asian countries), MASLD was associated with a 1.5‐ to 2‐fold increased risk of developing gastrointestinal cancers (e.g. stomach, pancreas, and colorectal cancers) and a 1.2‐ to 1.5‐fold increased risk of developing non‐GI cancers (e.g. lung, breast). All risks were independent of age, sex, smoking, obesity, diabetes, or other potential confounders.^5^

In Australia, the magnitude of risk of EHC in people with MASLD and the relationship with liver disease severity and metabolic comorbidity remains unclear.^6^, ^7^, ^8^ This information is important to inform cancer awareness programs and calculate its clinical and economic burden, given the high prevalence of MASLD (20–30%) in the general population. We estimated the cumulative age‐standardized incidence (ASI) of EHC in adults admitted to Queensland hospitals with MASLD with or without cirrhosis during 2007–2019 by sex and compared the observed incidence of cancers with that for the Queensland adult population. We also examined the association between the presence of cirrhosis and type 2 diabetes (T2D) and the development of EHC in the study cohort after adjusting for age and sex.

## Methods

We conducted a retrospective state‐wide cohort study of people hospitalized with MASLD during 2007–2019. We analyzed data from the Queensland Hospital Admitted Patient Data Collection (QHAPDC), death registrations from the Queensland Registry of Births, Deaths and Marriages, and the Queensland Cancer Register.

### Case selection

The selection of cases has been previously described.^9^ Briefly, MASLD was defined by at least one hospitalization with an International Classification of Diseases 10th edition – Australian Modification (ICD‐10‐AM) code for MASLD, MASH, or unspecified cirrhosis of liver.^10^ We excluded patients who ever had other liver diseases (e.g. alcohol‐related liver disease),^11^ and patients with a history of liver cancer, liver transplant, or liver disease decompensation prior to the first hospitalization with MASLD (index admission) during 2007–2019 (further details: Figure S1, Supporting information).

### Data sources

Sociodemographic and clinical data were obtained from QHAPDC (further details: Data S1). We ascertained the details of all cancers notified to the registry between 1 January 2007 and 31 December 2019 and diagnosed in patients included in the study.

### Outcome and study measurements

The primary outcomes were incident cancers notified to the Queensland Cancer Registry after the index admission. Cirrhosis and T2D status were assessed at index admission using ICD‐10‐AM codes for unspecified cirrhosis of liver (K74.6) and T2D (E11 to E14).

### Data analysis

All statistical analyses were performed in Stata 18.0 (StataCorp). We estimated cancer incidence by sex and cancer type for people aged ≥20 years. Incidence rates were age‐standardized to the Australian Standard Population and expressed per 100 000 person‐years. We reported ASI rate ratios (IRR) and corresponding 95% confidence interval (CI) and *P*‐value to compare ASI rates in the study cohort and the Queensland population.

Patients either had cancer or were censored at death or 31 December 2019. Multivariable Cox regression analysis of the MASLD cohort reported in terms of hazard ratios (HRs) with associated 95% CIs was used to examine the association between cirrhosis and T2D and the development of EHCs. Informed by a meta‐analysis of cohort studies that examined the risk of EHCs in people diagnosed with MASLD,^5^ cirrhosis, T2D, age, and sex were included in the model. A least absolute shrinkage and selection operators (LASSO) penalized regression Cox proportional hazards model was used to identify if there were other variables strongly associated with the development of EHCs. The final model included cirrhosis, T2D, age group, sex, and the interaction term cirrhosis‐T2D. Variables were checked to ensure that they adhered to the assumption of proportional hazards over time (Schoenfeld residuals). The vce(robust)option was used to obtain robust standard errors for the parameter estimates to control for mild violation of underlying assumptions.

## Results

We identified 11 483 subjects aged ≥20 years who were admitted to a Queensland hospital at least once with an ICD code for MASLD/MASH during Jul‐2007 to Dec‐2019 (Figure S1; *non‐cirrhotic N* = 9771 and *cirrhotic N* = 1712). Overall, they were mostly women (56.9%), ≥50 years (66.7%), Australian‐born (78.3%), resident of a major city (58.6%), and in most disadvantaged areas (64.7% in quintiles Q3 to Q5) (Table 1). Women were overrepresented in patients without cirrhosis compared with MASLD‐cirrhosis (58.5% *vs* 47.6%, *P* < 0.001) and older patients were overrepresented in patients with MASLD‐cirrhosis (89.3% were ≥50 years *vs* 62.7%, *P* < 0.001). A higher proportion of patients with MASLD‐cirrhosis (42.2%) had T2D compared to those without (31.0%, *P* < 0.001).

**Table 1:** Selected clinical and demographic characteristics of the cohort of patients with MASLD/MASH according to cirrhosis status at index hospital admission

	No cirrhosis	Cirrhosis	Total	*P*‐value
	*N* = 9771	*N* = 1712	*N* = 11 483	
Sex				
Male	4052 (41.5%)	897 (52.4%)	4949 (43.1%)	<0.001
Female	5719 (58.5%)	815 (47.6%)	6534 (56.9%)	
Age group				
20–29 years	606 (6.2%)	16 (0.9%)	622 (5.4%)	<0.001
30–39 years	1214 (12.4%)	53 (3.1%)	1267 (11.0%)	
40–49 years	1819 (18.6%)	113 (6.6%)	1932 (16.8%)	
50–59 years	2379 (24.3%)	307 (17.9%)	2686 (23.4%)	
60–69 years	2093 (21.4%)	466 (27.2%)	2559 (22.3%)	
70 years and over	1660 (17.0%)	757 (44.2%)	2417 (21.0%)	
Country of birth^†^				
Australia	7641 (78.6%)	1306 (76.5%)	8947 (78.3%)	<0.001
New Zealand, Oceania/Antarctica	572 (5.9%)	76 (4.5%)	648 (5.7%)	
Europe	952 (9.8%)	237 (13.9%)	1189 (10.4%)	
Africa & the Middle East	154 (1.6%)	26 (1.5%)	180 (1.6%)	
Asia	303 (3.1%)	42 (2.5%)	345 (3.0%)	
Americas	100 (1.0%)	20 (1.2%)	120 (1.0%)	
Indigenous status^‡^				
Non‐Indigenous	9193 (94.3%)	1621 (94.7%)	10 814 (94.4%)	0.50
Indigenous	552 (5.7%)	90 (5.3%)	642 (5.6%)	
Remoteness of residence				
Major city	5702 (58.4%)	1032 (60.3%)	6734 (58.6%)	0.24
Inner regional	2099 (21.5%)	362 (21.1%)	2461 (21.4%)	
Outer regional to very remote	1970 (20.2%)	318 (18.6%)	2288 (19.9%)	
Socioeconomic status				
Q1 most affluent	1683 (17.2%)	265 (15.5%)	1948 (17.0%)	0.096
Q2	1811 (18.5%)	299 (17.5%)	2110 (18.4%)	
Q3	1820 (18.6%)	307 (17.9%)	2127 (18.5%)	
Q4	2099 (21.5%)	401 (23.4%)	2500 (21.8%)	
Q5 most disadvantaged	2358 (24.1%)	440 (25.7%)	2798 (24.4%)	
Hospital sector				
Public	5051 (51.7%)	1041 (60.8%)	6092 (53.1%)	<0.001
Private or mix	4720 (48.3%)	671 (39.2%)	5391 (46.9%)	
Type 2 diabetes mellitus	3029 (31.0%)	722 (42.2%)	3751 (32.7%)	<0.001

^†^ Information missing for *N* = 54 (0.5%).

^‡^ Information missing for *N* = 27 (0.2%).

MASLD, metabolic dysfunction‐associated steatotic liver disease; MASH, metabolic dysfunction‐associated steatohepatitis.

### Incidence of cancer in the MASLD cohort

The median follow‐up from index admission to Dec‐2019 or death was 3.8 years (IQR 1.5–7.4 years; 54 204 person‐years). Totally 1104 new cases of primary cancer were diagnosed in 1018 patients (8.9% of the study cohort). The most commonly diagnosed cancers included prostate, melanoma, liver, lung, and colorectal in men, and breast, lung, melanoma, colorectal, and liver in women. The observed ASI of cancer (all cancers excluding nonmelanoma skin cancer) was 1459.9 per 100 000 person‐years (95% CI 1368.3–1557.1), 1668.2 per 100 000 person‐years in men (95% CI 1523.7–1827.4) and 1284.0 per 100 000 person‐years in women (95% CI 1169.6–1408.2) (Table 2).

**Table 2:** Age‐standardized incidence (ASI) rates per 100 000 person‐years of the most common cancers observed in the *whole study cohort* and in patients with MASLD/MASH *without cirrhosis* by sex compared to corresponding rates in the Queensland population age ≥20 years in 2016

	Study cohort (*N* = 11 483)				MASLD/MASH without cirrhosis (*N* = 9771)				Queensland population
	Cases	Incidence rate^†^ 95% CI	IRR (95% CI)		Cases	Incidence rate^†^ 95% CI	IRR (95% CI)		Incidence rate^†^ 95% CI
Male									
All cancers combined	568	1668.2 (1523.7–1827.4)	**1.94 (1.75–2.16)**	**<0.001**	413	1468.6 (1318.8–1635.4)	**1.71 (1.51–1.93)**	**<0.001**	859.2 (845.9–872.7)
Prostate	91	239.2 (190.4–304.0)	1.18 (0.90–1.54)	0.228	82	255.0 (199.6–328.5)	1.25 (0.95–1.66)	0.113	203.2 (196.9–209.7)
Melanoma	68	209.9 (160.4–276.4)	**1.65 (1.20–2.25)**	**0.002**	57	208.2 (154.9–280.7)	**1.63 (1.17–2.29)**	**0.004**	127.4 (122.3–132.7)
Liver	67	183.6 (140.4–243.5)	**12.08 (8.17–17.85)**	**<0.001**	19	57.6 (33.3–103.8)	**3.79 (1.91–7.51)**	**<0.001**	15.2 (13.5–17.0)
Lung	60	179.4 (134.9–241.1)	**2.22 (1.58–3.12)**	**<0.001**	42	158.6 (111.8–225.4)	**1.96 (1.31–2.93)**	**0.001**	80.9 (76.8–85.1)
Colorectal	49	150.4 (106.4–212.8)	**1.55 (1.05–2.30)**	**0.029**	38	144.5 (97.6–212.4)	1.49 (0.96–2.30)	0.073	97.0 (92.4–101.6)
Unknown primary	27	74.9 (48.2–120.7)	**3.90 (2.22–6.87)**	**<0.001**	15	48.9 (26.2–94.3)	**2.55 (1.21–5.37)**	**0.014**	19.2 (17.3–21.4)
Non‐Hodgkin lymphoma	22	58.7 (36.0–101.1)	**2.31 (1.26–4.25)**	**0.007**	15	47.4 (25.6–91.7)	1.87 (0.90–3.87)	0.094	25.4 (23.2–27.9)
Pancreas	22	56.6 (34.6–98.4)	**2.95 (1.57–5.53)**	**<0.001**	18	59.8 (33.8–107.9)	**3.11 (1.57–6.19)**	**0.001**	19.2 (17.2–21.3)
Stomach	17	57.3 (30.5–105.3)	**3.79 (1.81–7.94)**	**<0.001**	16	62.9 (33.2–116.1)	**4.17 (1.98–8.77)**	**<0.001**	15.1 (13.4–17.0)
Kidney	17	44.0 (25.0–83.6)	1.38 (0.70–2.74)	0.358	14	42.5 (22.6–85.3)	1.33 (0.63–2.81)	0.451	31.9 (29.4–34.6)
Female									
All cancers combined	536	1284.0 (1169.6–1408.2)	**1.99 (1.78–2.22)**	**<0.001**	419	1153.7 (1037.3–1281.1)	**1.79 (1.58–2.02)**	**<0.001**	646.1 (634.9–657.5)
Breast	101	215.0 (173.5–266.3)	1.21 (0.95–1.54)	0.129	90	221.8 (176.0–278.5)	1.25 (0.96–1.62)	0.096	177.9 (173.1–184.0)
Lung	44	104.5 (74.9–144.8)	**1.90 (1.29–2.81)**	**0.001**	37	110.3 (76.3–156.5)	**2.01 (1.32–3.05)**	**0.001**	54.9 (51.7–58.2)
Melanoma	43	95.1 (67.5–133.3)	1.13 (0.77–1.67)	0.532	34	85.2 (57.8–124.2)	1.01 (0.66–1.56)	0.949	84.0 (79.9–88.2)
Colorectal	41	106.6 (75.3–149.0)	**1.54 (1.04–2.29)**	**0.032**	32	99.1 (66.2–144.6)	1.43 (0.92–2.23)	0.113	69.2 (65.6–73.0)
Liver	36	91.9 (62.5–132.7)	**19.98 (10.99–36.31)**	**<0.001**	16	46.3 (25.6–79.9)	**10.07 (4.57–22.18)**	**<0.001**	4.6 (3.6–5.6)
Pancreas	29	68.8 (45.2–103.6)	**4.81 (2.82–8.20)**	**<0.001**	19	47.9 (27.6–80.7)	**3.35 (1.74–6.45)**	**<0.001**	14.3 (12.7–16.1)
Uterus	25	59.8 (36.3–95.2)	**2.60 (1.46–4.62)**	**0.001**	25	65.9 (40.4–104.1)	**2.87 (1.63–5.05)**	**<0.001**	23.0 (21.0–25.3)
Stomach	20	44.0 (26.3–73.1)	**4.89 (2.52–9.47)**	**<0.001**	11	26.5 (12.7–53.1)	**2.94 (1.24–7.00)**	**0.014**	9.0 (7.7–10.4)
Non‐Hodgkin lymphoma	19	42.1 (24.7–70.9)	**2.29 (1.21–4.31)**	**0.010**	12	33.5 (16.6–63.5)	1.82 (0.84–3.96)	0.131	18.4 (16.5–20.4)
Unknown primary	19	53.3 (31.6–87.0)	**4.59 (2.44–8.64)**	**<0.001**	11	37.5 (18.2–70.6)	**3.23 (1.45–7.22)**	**0.004**	11.6 (10.2–13.1)

^†^ Age standardized to the 2001 Australian standard population, and presented per 100 000 (males or females) including people age ≥20 years.

Bold values indicates statistically significance (*P* < 0.05).

MASLD, metabolic dysfunction‐associated steatotic liver disease; MASH, metabolic dysfunction‐associated steatohepatitis.

**Figure 1:**
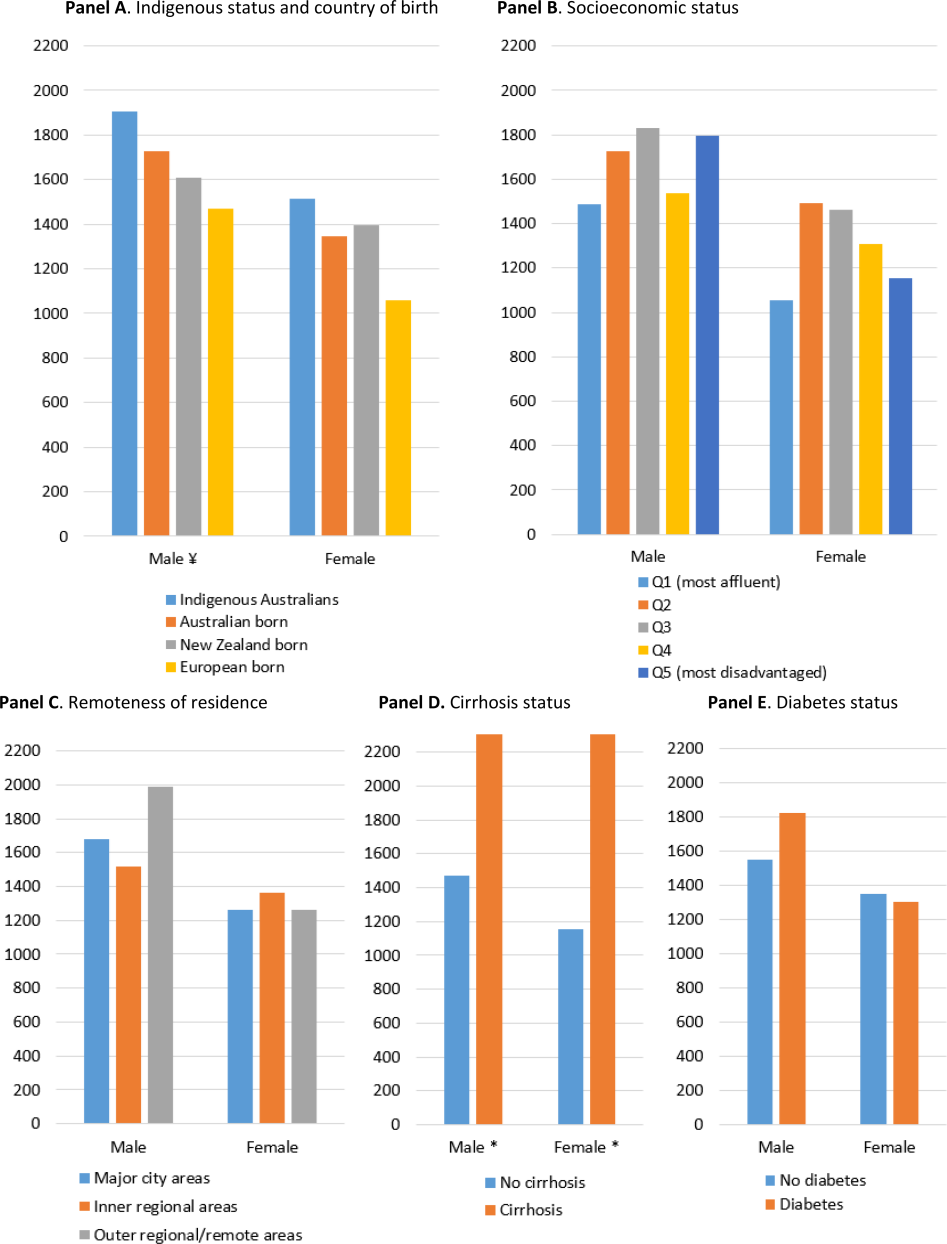
Age‐standardized incidence (ASI) rates per 100 000 person‐years of newly diagnosed cancers (all sites) observed in the cohort of patients with MASLD/MASH according to Indigenous status and country of birth (panel a), socioeconomic status (panel b), remoteness of residence (panel c), cirrhosis status (panel d), and diabetes status (panel e) at first admission with MASLD/MASH. MASLD, metabolic dysfunction‐associated steatotic liver disease; MASH, metabolic dysfunction‐associated steatohepatitis. *Indicates statistically significance (*P* < 0.05) according to cirrhosis status.

Among patients with MASLD/MASH without cirrhosis, the ASI of cancer (all cancers) was 1294.3 per 100 000 person‐years (95% CI 1200.5–1394.7). In patients with MASLD/MASH with cirrhosis, the ASI of cancer (all cancers) was 2532.8 per 100 000 person‐years (95% CI 2094.9–3103.1). Subsequent analyses were performed to compare and contrast ASI of cancer stratified by sociodemographic factors, liver disease severity and T2D in the study cohort. The ASI of cancer (all cancers) in the study cohort was similar across country of birth and Indigenous status, remoteness of residence, socioeconomic disadvantage, and presence of T2D at index admission for both men and women (Fig. 1 and Table S1). Compared to *non‐cirrhotic* MASLD/MASH, the ASI of cancer was 1.9‐fold higher in men (IRR = 1.92, 95% CI 1.32–2.79; *P* < 0.001) and 2.0‐fold higher in women (IRR = 2.01, 95% CI 1.29–3.13; *P* = 0.002) with MASLD‐cirrhosis.

### Higher incidence of cancers in patients with MASLD/MASH compared to the general population

**Figure 2:**
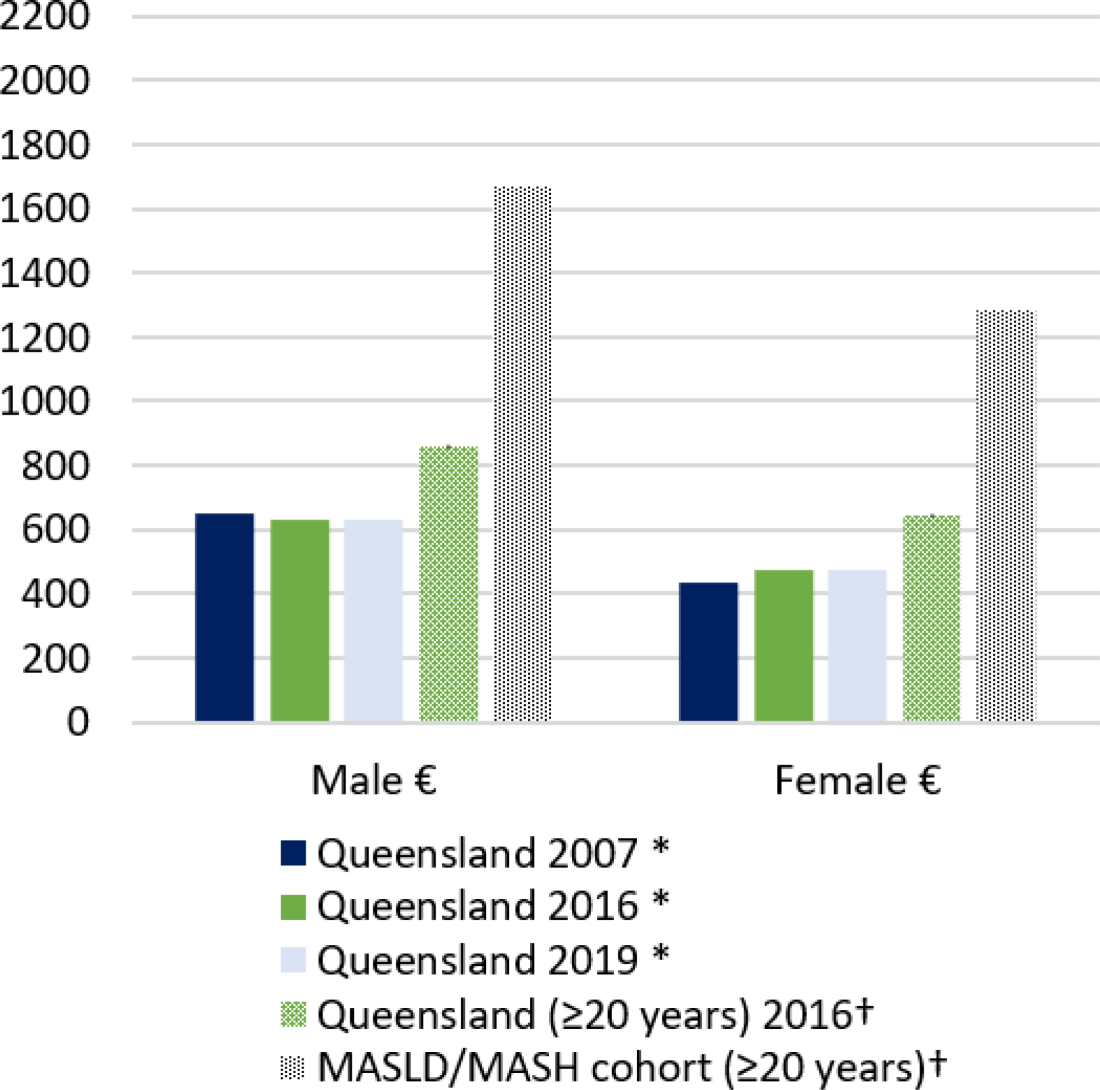
Age‐standardized incidence (ASI) rates per 100 000 person‐years of newly diagnosed cancers (all sites) observed in the whole cohort of patients with MASLD/MASH by sex compared to corresponding rates in the Queensland population in 2007, 2016 and 2019. *Age standardized to the 2001 Australian standard population, and presented per 100 000 persons (males or females) including all ages obtained from the Cancer Council Queensland. † Age standardized to the 2001 Australian standard population, presented per 100 000 person‐years (males or females) including people aged ≥20 years. **€** Indicates statistically significant (*P* < 0.05) when rate observed in the cohort of patients with MASLD/MASH is compared to rate in the Queensland population 2016 (age ≥20 years). MASLD, metabolic dysfunction‐associated steatotic liver disease; MASH, metabolic dysfunction‐associated steatohepatitis.

**Figure 3:**
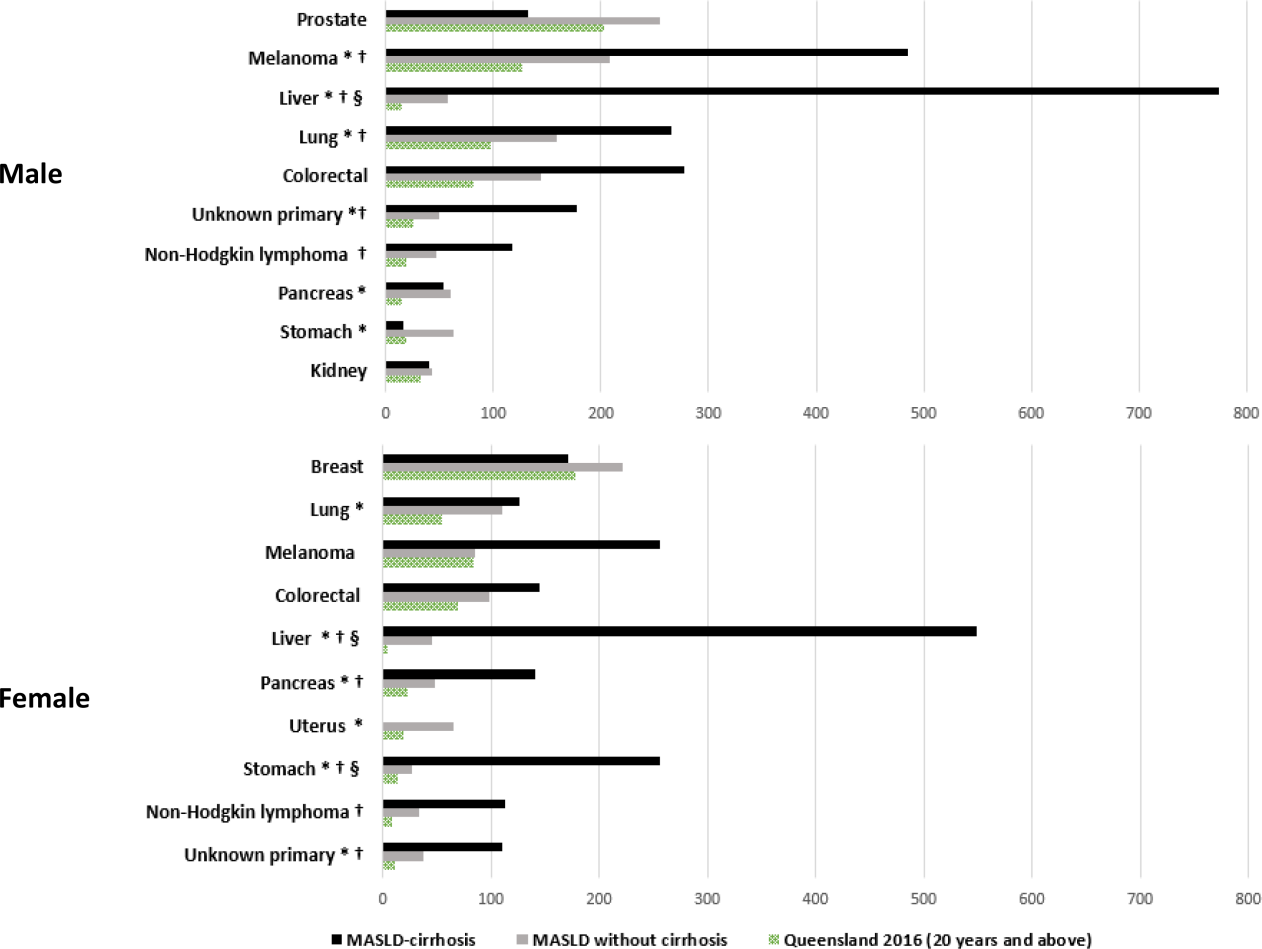
Age‐standardized incidence (ASI) rates per 100 000 person, years of the most common cancers observed in the cohort of patients with MASLD/MASH according to cirrhosis status and sex compared to corresponding rates in the Queensland population age ≥20 years in 2016. *Indicates statistically significant (*P* < 0.05) when comparing rates in the MASLD/MASH without cirrhosis cohort with that of the Queensland population. †Indicates statistically significant (*P* < 0.05) when comparing rates in the MASLD‐cirrhosis cohort with that of the Queensland population. § Indicates statistically significant (*P* < 0.05) when comparing rates in the MASLD‐cirrhosis cohort with that of the MASLD/MASH without cirrhosis cohort.

The ASI of cancer (all cancers) in the general Queensland population ≥20 years in 2016 was 859.2 per 100 000 person‐years in men and 646.1 per 100 000 person‐years in women. The corresponding incidence rate ratios comparing the incidence of cancer in the whole study cohort with that of the Queensland population were 1.94 for men (95% CI 1.75–2.16, *P* < 0.001) and 1.99 for women (95% CI 1.78–2.22, *P* < 0.001) (Table 2 and Fig. 2). As expected, the rates of liver cancer were over 12‐fold higher among men (IRR = 12.08, 95% CI 8.17–17.85, *P* < 0.001) and 20‐fold higher among women (IRR = 19.98, 95% CI 10.99, 36.31, *P* < 0.001) with MASLD/MASH compared to the general population. Incidence of stomach cancer, unknown primary, and pancreas in men and women were about 3‐ to 5‐fold higher compared to the general population. Of the most common cancers, cancers of the prostate and kidney in men and breast and melanoma in women were the only cancers with incidence comparable to the general population (Table 2 and Fig. 3).

Among patients with *non‐cirrhotic* MASLD/MASH, incidence of cancer (all cancers) was 1.71‐fold higher for men (95% CI 1.51–1.93, *P* < 0.001) and 1.79‐fold higher for women (95% CI 1.58–2.02, *P* < 0.001) compared to the general population. The rates of liver cancer were over 3.79‐fold higher among men (95% CI 1.91–7.51; *P* < 0.001) and 10.07‐fold higher among women (95% CI 4.57–22.18; *P* < 0.001) with MASLD/MASH compared to the general population (Table 2 and Fig. 3). The rates of melanoma, lung, unknown primary, pancreas and stomach in men and lung, pancreas, uterus, stomach, and unknown primary in women were significantly higher than corresponding rates in the general population with incidence rate ratios ranging from 1.6 to 4.2. Among patients with *MASLD‐cirrhosis*, incidence of cancer (all cancers) was 3.29‐fold higher for men (95% CI 2.48–4.36, *P* < 0.001) and 3.59‐fold higher for women (95% CI 2.52–5.11, *P* < 0.001), and the rates of liver cancer were over 50‐higher among men (IRR = 50.95, 95% CI 29.15–89.04; *P* < 0.001) and women (IRR = 119.50, 95% CI 32.18–443.80; *P* < 0.001) compared to the general population (Table S2).

### Examining the effect of cirrhosis and T2D on extrahepatic cancer risk in the MASLD cohort

Firstly, the ASI of EHC was stratified according to cirrhosis and T2D status. Incidence of EHC was 1.5‐fold higher in patients with cirrhosis (95% CI 1.13–2.00; *P* = 0.005) *versus* those without (Table 3). Of the most common EHCs, only stomach cancer had a higher incidence in women with cirrhosis (IRR = 9.67, 95% CI 1.26–74.04; *P* = 0.029) (Fig. 3; details about other EHCs in Table S3). When patients were stratified according to T2D status, there was no significant difference in incidence of EHCs (data on specific cancers not shown). Secondly, using Cox regression, we examined the association between the presence of cirrhosis and T2D and the development of EHCs in the study cohort (Table 4). In multivariable analysis, older age (e.g. ≥70 years adj‐HR = 4.59, 95% CI 3.61–5.83), male gender (adj‐HR = 1.20, 95% CI 1.05–1.37), and cirrhosis at index admission (adj‐HR = 1.37, 95% CI 1.11–1.70) were independently associated with EHC risk, while T2D was not.

**Table 3:** Age‐standardized incidence (ASI) rates per 100 000 person‐years of extrahepatic cancers according to cirrhosis and type 2 diabetes status among patients with MASLD/MASH

		Incidence rate ratio (95% CI; *P*‐value)		Incidence rate ratio (95% CI; *P*‐value)	
	Overall	Cirrhosis	No cirrhosis	T2D	No T2D
	Incidence rate 95% CI	Incidence rate 95% CI	Incidence rate 95% CI	Incidence rate 95% CI	Incidence rate 95% CI
Extrahepatic cancers					
*Male*	1484.6 (1347.6–1636.5)	2050.7 (1472.1–2929.1)	1411.0 (1263.7–1575.3)	1632.2 (1398.3–1991.5)	1369.8 (1206.9–1553.0)
*N* = 501		IRR = 1.45 (0.92–2.29; *P* = 0.107)		IRR = 1.19 (0.88–1.61; *P* = 0.257)	
*Female*	1192.1 (1082.3–1311.7)	1769.4 (1350.5–2596.4)	1107.4 (993.4–1232.4)	1213.4 (927.8–1579.2)	1256.5 (1115.6–1411.5)
*N* = 500		**IRR = 1.60 (1.03–2.47; *P* = 0.035)**		IRR = 0.97 (0.66–1.42; *P* = 0.858)	
*Persons*	1325.6 (1238.3–1418.7)	1867.4 (1533.7–2334.6)	1243.4 (1151.3–1342.1)	1413.4 (1204.0–1664.8)	1308.8 (1201.3–1424.4)
*N* = 1001		**IRR = 1.50 (1.13–2.00; *P* = 0.005)**		IRR = 1.08 (0.84–1.38; *P* = 0.542)	

Bold values indicate statistically significance (*P* < 0.05) according to cirrhosis status.

*N*, number; IRR, incidence rate ratio; CI, confidence interval; T2D, type 2 diabetes.

**Table 4:** Results from Cox regression analyses examining the association between the presence of cirrhosis and type 2 diabetes and the development of extrahepatic cancers among patients with MASLD/MASH

		Hazard ratio (95%CI)	Adjusted hazard ratio (95%CI)^†^
Sex	Male (*vs* female)	**1.30 (1.14–1.48)**	**1.20 (1.05–1.37)**
Age group	<50 years	Reference	Reference
	50–65 years	**4.00 (3.22–4.97)**	**3.81 (3.06–4.76)**
	70 years and over	**5.07 (4.03–6.39)**	**4.59 (3.61–5.83)**
Country of birth^‡^	Overseas born (*vs* Australia)	0.91 (0.78–1.07)	N/S
Indigenous status^§^	Indigenous (*vs* non‐Indigenous)	0.80 (0.59–1.09)	N/S
Remoteness of residence	Major city	Reference	N/S
	Inner regional	1.05 (0.90–1.23)	
	Outer regional to very remote	0.89 (0.75–1.06)	
Socioeconomic status	Q1 most affluent	Reference	N/S
	Q2	1.25 (1.00–1.58)	
	Q3	1.40 (1.12–1.74)	
	Q4	1.18 (0.95–1.48)	
	Q5 most disadvantaged	**1.26 (1.02–1.57)**	
Hospital sector	Private or mix (*vs* public)	1.03 (0.91–1.18)	N/S
Cirrhosis		**1.68 (1.43–1.97)**	**1.37 (1.11–1.70)**
Type 2 diabetes mellitus		**1.33 (1.17–1.52)**	1.11 (0.96–1.29)

^†^ Model included cirrhosis, type 2 diabetes, age group, sex, and the interaction term cirrhosis‐type 2 diabetes

^‡^ Information missing for *N* = 54 (0.5%).

^§^ Information missing for *N* = 27 (0.2%).

Bold values indicate statistically significance (*P* < 0.05). Variable not selected (N/S) for inclusion in the model.

CI, confidence interval.

## Discussion

We found that people living with MASLD have a 2‐fold higher incidence of cancer (all cancers) than the general Queensland population. As expected, the rates of liver cancer were more than 12‐fold and 20‐fold higher among men and women with MASLD/MASH, respectively, compared to the general population. Importantly, although less widely recognized, the rates of cancer of the stomach, pancreas, and unknown primary in both men and women were about 3‐ to 5‐fold higher compared to the general population. In fact, of the most common cancers, prostate, and kidney in men and breast and melanoma in women were the only cancers with incidence comparable to the general population.

Our Australian data are similar to the findings from many international studies that show an increased risk of incident EHC. In a US population, people with MASLD had a nearly 2‐fold increase in the overall risk of incident cancers (particularly liver, uterine, stomach, pancreas and colon) when compared to an age‐ and sex‐matched general population cohort.^2^ Among participants in the UK Biobank (2006–2019), individuals at high risk for MASLD had a 1.23‐fold increased risk of non‐liver gastrointestinal cancers compared with individuals at low risk for MASLD.^3^ In contrast, a smaller matched cohort study from a single US center (1412 subjects, 34% with MASLD) did not show an increased risk for EHC after matching major risk factors including age, sex, race, BMI, and diabetes status.^4^ As MASLD is a multisystem disorder, it remains difficult to investigate its contribution to EHC independently of obesity and metabolic dysfunction.^12^

In our study, the ASI of cancer (all cancers) was not influenced by the presence of T2D at index admission. The impact of obesity could not be assessed, as ICD‐10‐AM codes have a very low sensitivity for identifying obesity.^13^ Both obesity and T2D are associated with increased risk for some common cancers and potential biological mechanisms (including the insulin/insulin‐like growth factor axis, hyperglycemia, chronic inflammation, sex hormones, and an altered microbiome) may also underlie the relationship between MASLD and EHC.^14^, ^15^ This underscores the need for an integrated approach to managing metabolic dysregulation and supports the shift away from “disease silos” towards holistic models of care for multisystem diseases like MASLD, in order to address the increased risk of cancer. Of the EHC with an increased incidence in people with MASLD, colorectal cancer can be identified by screening, emphasizing the importance of engaging these subjects in the national bowel cancer screening program.

Our data confirm that *non‐cirrhotic* MASLD is a risk factor for primary liver cancer. As expected, the rates of liver cancer increased markedly in the setting of cirrhosis, largely accounting for the 2‐fold increase in cancer incidence between people with MASLD with and without cirrhosis. In contrast, except for stomach cancer in women, the incidence of EHC was similar in patients with MASLD in the presence or absence of cirrhosis. However, after adjusting for age, sex, T2D, and the interaction between T2DM and cirrhosis, there was 1.37‐fold increase in risk of EHC in people with MASLD‐cirrhosis compared with those without cirrhosis. Older age and male gender, but not T2D, were also independently associated with development of EHC. These findings suggest that insulin resistance is not a primary mediator between MASLD and EHC. The relationship between liver disease severity and EHC may be confounded by other metabolic risk factors such as obesity, that are linked to both severity of MASLD and increased cancer risk. In fact, a meta‐analysis of the relationship between EHC and MASLD found that EHC rates were not higher in patients with advanced fibrosis or cirrhosis.^12^

MASLD is an underrecognized public health concern in Australia, with overall incidence and mortality rates through hepatic and non‐hepatic complications continuing to rise.^16^ Liver‐related outcomes associated with MASLD are well‐recognized, and these individuals are at risk of progression to cirrhosis, end‐stage liver disease, and HCC.^9^, ^16^ With the rising incidence of HCC in Australia, HCC surveillance in people with cirrhosis is widely implemented. Importantly, our data show that the vast majority of cancers occurring in people with MASLD (88.2% in men and 93.3% in women) are due to EHC and are not linked to liver disease severity. To date, most guidelines for the management of MASLD do not discuss screening strategies for EHC, as the cost‐effectiveness of additional cancer screening has not been evaluated. Due to the high prevalence of MASLD in the general population, there is concern that EHC may become a substantial health and economic issue,^12^ and people with MASLD should be encouraged to engage in relevant national cancer screening programs.

### Strengths and limitations

As the notification of cancer is a legal requirement for all hospitals and pathology services in Australia, we have a near complete documentation of cancers, removing the possibility of selection bias. The study included a population‐based sample of patients with MASLD ascertained using strict criteria for case identification.^11^, ^17^ However identification of MASLD/MASH through ICD codes is likely to underestimate its prevalence,^13^ and the study did not identify all patients with MASLD in the general population, particularly those who are managed in primary care and do not have decompensated cirrhosis or comorbidities that warranted hospitalization. The incidence of cancer in a population with a lesser burden of comorbidity may differ from that of the study population. The small number of cases for some cancers meant confidence intervals were wide, and some cancer‐specific results should be interpreted with due caution. The QHAPDC data are inadequate for assessing the severity of liver disease, and lack of data of liver fibrosis is a study limitation as fibrosis stage is an important predictor of an individual's prognosis.^18^, ^19^ Importantly, a median of 3.8 years of follow‐up may not be sufficient to fully assess incidence of cancer among people with MASLD. Moreover, the definition of NAFLD was used to identify and refer to MASLD. Several reanalyses from existing data support that NAFLD‐related findings can be fully extrapolated to individuals with MASLD.^20^ For example, evidence from the US population^21^ showed that patients captured by the definition of MASLD were also covered by the definition of NAFLD, and 5% of patients with NAFLD were not captured by the definition of MASLD as they did not have the cardiometabolic risk factors that are required to meet the MASLD definition. For the latter group, NAFLD patients were younger and without cardiometabolic risks, likely explaining the slightly higher mortality among US patients with MASLD than that of patients with NAFLD. If we had included the presence of metabolic risks in our definition of MASLD, the likely effect would be a slightly older cohort and therefore with an even higher incidence of cancer than the general Queensland population. Future analyses of population‐based data on this study cohort, with longer follow‐up, and using a definition that fully captures MASLD patients, could address these study limitations.

## Conclusion

The 2‐fold increase in incidence of cancer in the Australian population with MASLD raises concern about its future clinical and economic burden given the high prevalence of MASLD (20–30%) in the general population and reinforces the need for a holistic approach to identification and management of this multisystem metabolic disorder. Our findings will help raise awareness among clinicians about the importance of cancer vigilance in this patient group. A diagnosis of MASLD provides an opportunity to address metabolic risk factors and engage people with MASLD in the available national colorectal cancer screening program.

## Consent to participate

De‐identifiable data without consent was included. The study was approved by the Metro South Health Services and QIMR Berghofer Human Research Ethics Committees (HREC/17/QPAH/23; P2209). All procedures followed were in accordance with the ethical standards of the abovementioned committees and with the Helsinki Declaration of 1975, as revised in 2008.

## Supporting information


**Data S1.** Supporting Information.

## Data Availability

The data that support the findings of this study contain potentially sensitive and/or identifying information that could compromise the privacy of the participants. Therefore, data are not publicly available. Data may, however, be available from the authors upon reasonable request with approval from relevant ethics committees.
