# Supplementary material for: Australians with metabolic dysfunction‐associated steatotic liver disease have a twofold increase in the incidence of cancer
Source: JGH Open. 2024 Jul 22;8(7):e70000. doi: 10.1002/jgh3.70000 (PMC11261590; doi:10.1002/jgh3.70000)
Supplement: Supplementary file 1 — Data S1. Supporting Information. [file JGH3-8-e70000-s001.docx]

**Supporting Information**

**Supplementary methods and results**

**Case selection**

In 2023, a global expert consensus statement recommended that the currently available ICD codes for non-alcoholic

fatty liver disease (NAFLD) and non-alcoholic steatohepatitis (NASH) be used to define metabolic-dysfunction associated steatotic liver disease (MASLD) and metabolic-dysfunction–associated steatohepatitis (MASH), respectively.^1^ In this study, MASLD was defined^2^ by at least one hospitalisation with an International Classification of Diseases 10^th^ edition – Australian Modification (ICD-10-AM) code for NAFLD (K76.0), NASH (K75.8) or unspecified cirrhosis of liver (K74.6). As Hagstrom et al^2^ and Petta et al,^3^ and our previous analysis of this study cohort,^4^ we excluded patients who ever had other liver diseases (alcohol-related liver disease, chronic viral hepatitis, autoimmune liver disease, hemochromatosis, Wilson’s disease, alpha-1-antitrypsin deficiency, Budd-Chiari syndrome, chronic hepatitis, unspecified, secondary/unspecified biliary cirrhosis), people whose age or residential location at admission was unknown or outside Queensland, and patients with a recorded diagnosis of liver cancer prior to the index admission, and with a history of liver transplant, or liver disease decompensation prior to the index admission (**Supplementary Figure 1**).

**Supplementary Figure 1**. Flow chart for case ascertainment


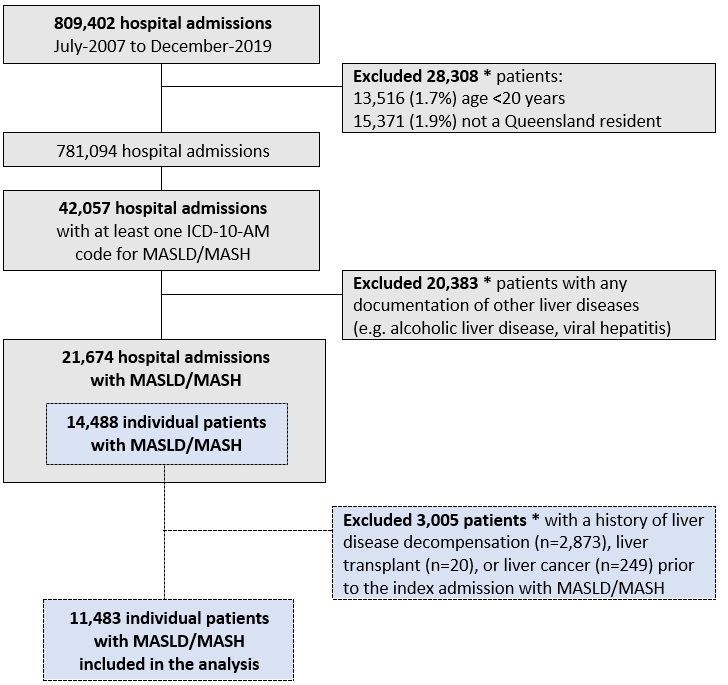


*Patients may have more than one exclusion criteria***Data sources***

The Statistical Analysis Linkage Unit of the Queensland Department of Health undertook dataset linkage. Sociodemographic data items from QHAPDC included: age group at index admission (categorized by 5-year age groups), sex, country of birth (the region in which a person was born according to the Standard Australian Classification of Countries),^5^ Indigenous status (based on self-identification as either Aboriginal only, Aboriginal and Torres Strait Islander or Torres Strait Islander only), and residential location at the time of index admission (categorized according to the level of remoteness of residence based on the Accessibility/Remoteness Index of Australia (ARIA+)^6^ and the Index of Relative Socioeconomic Advantage and Disadvantage (IRSAD).^7^ Presence of cirrhosis at index admission was based on recorded ICD‐10‐AM codes for primary or other diagnosis for ‘other and unspecified cirrhosis of liver’ (K74.6). Type 2 diabetes (T2D) at the time of hospitalization was measured using ICD codes listed in the Charlson Comorbidity Index.^8^ Date of death was obtained from the Queensland Registry of Births, Deaths and Marriages.

We ascertained the details of all cancers notified to the Queensland Cancer Register between 1 January 2007 and 31 December 2019 (inclusive) and diagnosed in patients included in the study cohort. The cancer registry only records primary site diagnosis that are considered new primary sites according to various multiple primary site cancer rules followed by all Australian cancer registries. These rules include recording synchronous tumours with different stage, same site different histological type, contralateral tumours of bilateral sites (e.g. breast left and right), different site and same morphology (e.g. melanomas) or different site and different histological type (e.g. tongue squamous cell carcinoma and liver hepatocellular carcinoma).

**References**

**1.** Hagstrom H, Adams LA, Allen AM, et al. The future of International Classification of Diseases coding in steatotic liver disease: An expert panel Delphi consensus statement. **Hepatol Commun***.* Feb 1 2024;8(2).

**2.** Hagstrom H, Adams LA, Allen AM, et al. Administrative Coding in Electronic Health Care Record-Based Research of NAFLD: An Expert Panel Consensus Statement. **Hepatology***.* Jul 2021;74(1):474-482.

**3.** Petta S, Ting J, Saragoni S, et al. Healthcare resource utilization and costs of nonalcoholic steatohepatitis patients with advanced liver disease in Italy. **Nutr Metab Cardiovasc Dis***.* Jun 9 2020;30(6):1014-1022.

**4.** O'Beirne J, Skoien R, Leggett BA, et al. Diabetes mellitus and the progression of non-alcoholic fatty liver disease to decompensated cirrhosis: a retrospective cohort study. **Med J Aust***.* Oct 16 2023;219(8):358-365.

**5.** Australian Bureau of Statistics (ABS). *1269.0 - Standard Australian Classification of Countries (SACC), 2016* Canberra: ABS; 2016.

**6.** Australian Institute of Health and Welfare (AIHW). *Rural, regional and remote health: A guide to remoteness classifications.* Canberra, Australia: AIHW; 2004 https://www.aihw.gov.au/reports/rural-remote-australians/guide-to-remoteness-classifications/summary.

**7.** Australian Bureau of Statistics (ABS). *Census of Population and Housing: Socio-economic Indexes for Areas (SEIFA), Australia, 2006.* Canberra, Australia: ABS; 2008 https://www.abs.gov.au/ausstats/abs@.nsf/mf/2033.0.55.001.

**8.** Charlson ME, Pompei P, Ales KL, MacKenzie CR. A new method of classifying prognostic comorbidity in longitudinal studies: development and validation. **J Chronic Dis***.* 1987;40(5):373-383.

**9.** Australian Institute of Health and Welfare (AIHW). *Cancer in Australia 2021.* Canberra: AIHW; 2021.

**10.** Australian Bureau of Statistics (ABS). *Population by age and sex - 2001 census edition.* Canberra: ABS; 2003. ABS Cat. No. 3201.0.

**Supplementary Table 1**. Age-standardised incidence rates per 100,000 person, years of newly diagnosed cancers (all sites) observed in the cohort of patients with MASLD/MASH with or without cirrhosis by sex, and subgroup analysis of different patient populations

|  | **Cases** | **Incidence rate**† **95%CI** |
| --- | --- | --- |
| **Male** |  |  |
| Queensland population 2007 ***** | 12,978 | 653.6 N/A |
| Queensland population 2016 ***** | 16,391 | 629.8 N/A |
| Queensland population 2019 ***** | 18,325 | 639.2 N/A |
| Queensland population 2016 **†** (age ≥20 years) | 16,225 | 859.2 (845.9-872.7) |
| Overall study cohort 2007-2019 (age ≥20 years) | 568 | 1,668.2 (1,523.7-1,827.4) |
| Indigenous Australians | 18 | 1,906.4 (1,050.5-3,331.2) |
| Country of birth |  |  |
| Australia | 449 | 1,729.5 (1,560.9-1,916.9) |
| New Zealand, Oceania and Antarctica | 23 | 1,607.2 (912.7 2,707.1) |
| Europe | 73 | 1,471.1 (1,081.9-3,458.2) |
| Africa and the Middle East | 10 | 2,245.9 (1,001.3-4,410.7) |
| Asia | ≤ 5 **¥** | 1,126.3 (317.3-2,742.6) |
| Remoteness of residence |  |  |
| Major city areas | 338 | 1,675.9 (1,488.1-1,888.3) |
| Inner regional areas | 130 | 1,517.7 (1,234.5-1,882.4) |
| Outer regional areas | 100 | 1,989.5 (1,594.1-2,491.0) |
| Socioeconomic status (quintiles) |  |  |
| Q1 (most affluent) | 73 | 1,490.0 (1,142.1-1,925.5) |
| Q2 | 103 | 1,725.1 (1,392.0-2,138.7) |
| Q3 | 120 | 1,829.1 (1,490.0-2,273.1) |
| Q4 | 119 | 1,539.1 (1,245.2-1,967.8) |
| Q5 (most disadvantaged) | 153 | 1,797.1 (1,499.0-2,161.7) |
| Cirrhosis status **£** |  |  |
| MASLD/MASH only (no cirrhosis) | 413 | 1,468.6 (1,318.8-1,635.4) |
| MASLD-cirrhosis | 155 | 2,825.1 (2,193.4-3,734.0) |
| Type 2 diabetes mellitus status |  |  |
| No diabetes | 322 | 1548.9 (1375.8-1742.2) |
| Diabetes | 246 | 1821.4 (1576.7-2189.0) |
| **Female** |  |  |
| Queensland population 2007 ***** | 9,508 | 437.4 N/A |
| Queensland population 2016 ***** | 13,126 | 474.4 N/A |
| Queensland population 2019 ***** | 14,341 | 477.2 N/A |
| Queensland population 2016 **†** (age ≥20 years) | 12,990 | 646.1 (634.9-657.5) |
| Overall study cohort 2007-2019 (age ≥20 years) | 536 | 1,284.0 (1,169.6-1,408.2) |
| Indigenous Australians | 27 | 1,515.4 (909.8-2,350.1) |
| Country of birth |  |  |
| Australia | 427 | 1,346.0 (1,212.6-1,491.7) |
| New Zealand, Oceania and Antarctica | 27 | 1,396.9 (873.6-2,175.3) |
| Europe | 61 | 1,059.9 (787.2-3,696.7) |
| Africa and the Middle East | ≤ 5 **¥** | 499.6 (61.7-3,582.2) |
| Asia | 10 | 1,004.5 (458.3-2,288.2) |
| Remoteness of residence |  |  |
| Major city areas | 319 | 1,260.0 (1,111.6-1,426.3) |
| Inner regional areas | 127 | 1,360.4 (1,114.9-1,652.7) |
| Outer regional areas | 90 | 1,265.9 (1,005.5-1,579.5) |
| Socioeconomic status (quintiles) |  |  |
| Q1 (most affluent) | 82 | 1,053.9 (830.2-1,342.4) |
| Q2 | 100 | 1,491.7 (1,177.1-1,870.2) |
| Q3 | 119 | 1,460.7 (1,199.1-1,787.5) |
| Q4 | 113 | 1,306.5 (1,063.6-1,595.2) |
| Q5 (most disadvantaged) | 122 | 1,156.3 (949.2-1,404.9) |
| Cirrhosis status **£** |  |  |
| MASLD/MASH only (no cirrhosis) | 419 | 1,153.7 (1,037.3-1,281.1) |
| MASLD-cirrhosis | 117 | 2,319.1 (1,698.8-3,329.0) |
| Type 2 diabetes mellitus status |  |  |
| No diabetes | 344 | 1,346.0 (1,199.6-1,506.6) |
| Diabetes | 192 | 1,303.2 (1,013.0-1,671.6) |

Note: Metabolic dysfunction-associated steatotic liver disease (MASLD);

Metabolic dysfunction-associated steatohepatitis (MASH);

***** Number of cases and age standardised to the 2001 Australian standard population,^9^ presented per 100,000 person, years (males or females) including all ages (95%CI was not available (N/A);

**†** Age standardised to the 2001 Australian standard population, and presented per 100,000 (males or females) including people age ≥20 years;

**¥** Exact number not reported due to privacy issues;

**£** Indicates statistically significance (p<0.05) according to cirrhosis status;

**Supplementary Table 2**. Age-standardised incidence rates per 100,000 person-years of the most common cancers observed in the cohort of patients with *MASLD/MASH with cirrhosis* comparing to corresponding rates in the Queensland population age ≥20 years in 2016

|  |  | **Cases** | **Incidence rate**† (**95%CI)** |  | **IRR (95%CI)** | **p-value** |
| --- | --- | --- | --- | --- | --- | --- |
| **Male** |  |  |  |  |  |  |
| All cancers combined |  | 155 | 2,825.1 (2,193.4-3,734.0) |  | **3.29 (2.48-4.36)** | **<0.001** |
| Prostate |  | 9 | 132.6 (60.4-682.6) |  | 0.65 (0.19-2.26) | 0.501 |
| Melanoma |  | 11 | 485.0 (122.2-1,325.1) |  | **3.81 (1.11-13.06)** | **0.034** |
| Liver |  | 48 | 774.4 (552.4-1,339.9) |  | **50.95 (29.15-89.04)** | **<0.001** |
| Lung |  | 18 | 264.7 (154.8-801.8) |  | **3.27 (1.37-7.84)** | **0.008** |
| Colorectal |  | 11 | 278.2 (81.8-908.4) |  | 2.87 (0.82-10.02) | 0.099 |
| Unknown primary |  | 12 | 177.7 (89.8-722.2) |  | **9.26 (2.93-29.19)** | **<0.001** |
| Non-Hodgkin lymphoma |  | 7 | 117.5 (44.5-674.2) |  | **4.63 (1.08-19.75)** | **0.039** |
| Pancreas |  | ≤ 5 **¥** | 54.4 (14.6-623.6) |  | 2.83 (0.39-20.61) | 0.304 |
| Stomach |  | ≤ 5 **¥** | 15.5 (0.40-600.1) |  | 1.03 (0.02-44.78) | 0.989 |
| Kidney |  | ≤ 5 **¥** | 40.3 (8.10-614.6) |  | 1.26 (0.13-11.94) | 0.838 |
| **Female** |  |  |  |  |  |  |
| All cancers combined |  | 117 | 2,319.1 (1,698.8-3,329.0) |  | **3.59 (2.52-5.11)** | **<0.001** |
| Breast |  | 11 | 171.9 (80.6-927.2) |  | 0.97 (0.28-3.38) | 0.957 |
| Lung |  | 7 | 127.3 (47.4-897.6) |  | 2.32 (0.50-10.71) | 0.281 |
| Melanoma |  | 9 | 256.5 (75.5-1,054.1) |  | 3.05 (0.78-11.99) | 0.110 |
| Colorectal |  | 9 | 145.2 (62.1-908.2) |  | 2.10 (0.52-8.46) | 0.298 |
| Liver |  | 20 | 549.7 (170.2-1,509.1) |  | **119.50 (32.18-443.80)** | **<0.001** |
| Pancreas |  | 10 | 141.3 (67.4-901.6) |  | **9.88 (2.40-40.69)** | **0.002** |
| Uterus |  | nil | - |  | **-** | **-** |
| Stomach |  | 9 | 256.3 (75.2-1,054.1) |  | **28.48 (6.54-123.91)** | **<0.001** |
| Non-Hodgkin lymphoma |  | 7 | 113.6 (40.3-887.7) |  | **6.17 (1.18-32.22)** | **0.031** |
| Unknown primary |  | 8 | 111.2 (47.6-881.9) |  | **9.59 (1.97-46.76)** | **0.005** |

Note: Age-standardised incidence rate-ratio (IRR);

Metabolic dysfunction-associated steatotic liver disease (MASLD);

Metabolic dysfunction-associated steatohepatitis (MASH);

Bold values indicates statistically significance (p<0.05);

† Age standardised to the 2001 Australian standard population,^10^ and presented per 100,000 (males or females) including people age ≥20 years;

**¥** Exact number not reported due to privacy issues

**Supplementary Table 3**. Incidence rate ratio comparing age-standardised incidence rates of the most common cancers observed in patients with MASLD-cirrhosis vs MASLD/MASH without cirrhosis

|  |  | **IRR (95%CI)** | **p-value** |
| --- | --- | --- | --- |
| **Male** |  |  |  |
| Prostate |  | 0.52 (0.12-2.24) | 0.381 |
| Melanoma |  | 2.33 (0.53-10.33) | 0.266 |
| Liver |  | **13.44 (4.89-36.97)** | **<0.001** |
| Lung |  | 1.67 (0.52-5.39) | 0.392 |
| Colorectal |  | 1.93 (0.39-9.46) | 0.420 |
| Unknown primary |  | 3.63 (0.68-19.55) | 0.133 |
| Non-Hodgkin lymphoma |  | 2.48 (0.34-18.26) | 0.373 |
| Pancreas |  | 0.91 (0.08-10.62) | 0.940 |
| Stomach |  | 0.25 (0.00-17.85) | 0.521 |
| Kidney |  | 0.95 (0.06-16.05) | 0.971 |
| **Female** |  |  |  |
| Breast |  | 0.78 (0.18-3.31) | 0.731 |
| Lung |  | 1.15 (0.19-7.19) | 0.878 |
| Melanoma |  | 3.01 (0.55-16.49) | 0.204 |
| Colorectal |  | 1.47 (0.26-8.28) | 0.666 |
| Liver |  | **11.87 (2.26-62.46)** | **0.003** |
| Pancreas |  | 2.95 (0.47-18.45) | 0.247 |
| Uterus |  | - | - |
| Stomach |  | **9.67 (1.26-74.04)** | **0.029** |
| Non-Hodgkin lymphoma |  | 3.39 (0.37-31.13) | 0.280 |
| Unknown primary |  | 2.97 (0.35-25.14) | 0.319 |

Note: Age-standardised incidence rate-ratio (IRR);

Metabolic dysfunction-associated steatotic liver disease (MASLD);

Metabolic dysfunction-associated steatohepatitis (MASH);

Bold values indicates statistically significance (p<0.05);
